# Case report: Rhabdomyolysis and kidney injury in a statin-treated hypothyroid patient–kill two birds with one stone

**DOI:** 10.3389/fmed.2022.1046330

**Published:** 2022-11-10

**Authors:** Wen-Fang Chiang, Jenq-Shyong Chan, Po-Jen Hsiao, Shih-Hua Lin

**Affiliations:** ^1^Division of Nephrology, Department of Medicine, Armed Forces Taoyuan General Hospital, Taoyuan, Taiwan; ^2^Division of Nephrology, Department of Medicine, National Defense Medical Center, Tri-Service General Hospital, Taipei, Taiwan

**Keywords:** acute kidney injury, cholesterol, hypothyroidism, hydroxymethylglutaryl-CoA reductase inhibitors, rhabdomyolysis

## Abstract

Statin treatment for hypercholesterolemia may cause reversible rhabdomyolysis and acute kidney injury in susceptible patients. However, persistent rhabdomyolysis and acute kidney injury following discontinuation of statins require careful evaluation of the underlying causes to avoid missing a curable disease. We describe a 50-year-old woman with hypercholesterolemia [total cholesterol 345 mg/dl, low-density lipoprotein cholesterol (LDL-C) 266 mg/dL] on atorvastatin therapy (40 mg daily) for 1 month that presented with myalgia and muscle weakness. Relevant laboratory studies revealed persistent higher hypercholesterolemia with total cholesterol (312 mg/dL), high creatine kinase (CK) (5,178 U/L), and high creatinine levels (1.5 mg/dL) without dysmorphic red blood cells and proteinuria. Despite the cessation of statin therapy, serum CK level increased to 9,594 U/L, and creatinine remained at 1.5 mg/dL. A thorough work-up to assess potential underlying causes indicated low T3 and free T4 and high thyroid-stimulating hormone (TSH) levels, consistent with hypothyroidism. With aggressive thyroxine replacement for 1 month, all of the clinical features, along with elevated serum CK and creatinine levels, were completely resolved. This case highlights the fact that hypothyroidism must be kept in mind as a potential cause of concomitant myopathy and kidney injury, especially in patients with statin-resistant hypercholesterolemia.

## Introduction

Statins, 3-hydroxy-3-methylglutaryl coenzyme A (HMG-CoA) reductase inhibitors, can effectively treat hypercholesterolemia and reduce the occurrence of cardiovascular events. Although statins are well-tolerated in most patients, they may cause various musculoskeletal complications, so-called statin-induced myopathy ranging from myalgia, myositis, and mild creatine kinase (CK) elevations to flank rhabdomyolysis with serum CK levels exceeding 10 times the upper limit of normal. The overall risk of statin-induced rhabdomyolysis is low (1 case/10,000 person-years of treatment), and the occurrence of acute kidney injury complicated with rhabdomyolysis is rare (1). Furthermore, rhabdomyolysis with acute kidney injury is usually reversible following statin discontinuation. In this report, we describe a statin-resistant hypercholesterolemic patient who manifested flank rhabdomyolysis and acute kidney injury, which are not caused by stain-induced myopathy but unnoticed hypothyroidism. A comparison between statin and hypothyroidism-related myopathy was also made.

## Case description

A 50-year-old woman presented to the hospital with a 1-week history of muscle weakness and pain in the extremities. She began experiencing fatigue several months before the presentation; however, she attributed it to menopause. One month before the presentation, she was seen by a local doctor with complaints of increasing fatigue. Laboratory data showed a serum creatinine level of 0.9 mg/dL. Her lipid profile revealed a triglyceride level of 244 mg/dL, a total cholesterol level of 345 mg/dL, a low-density lipoprotein cholesterol (LDL-C) level of 266 mg/dL, and a high-density lipoprotein cholesterol (HDL-C) of 69 mg/dL. Therefore, atorvastatin 40 mg daily was initiated. Her medical history was unremarkable. The patient did not perform vigorous physical exercise or consume alcohol or any other drugs. There was no family history of thyroid or muscle diseases, hypercholesterolemia, or premature cardiovascular disease.

On physical examination, the patient was of average build, and her weight was 75 kg. Blood pressure was 150/94 mm Hg, pulse rate was regular 76 beats/min, respiratory rate was 17 breaths/min, and temperature was 36.2°C. The thyroid gland was normal in size. Muscle strength in the proximal limbs was diminished, and there was slow relaxation of deep tendon reflexes. No fasciculation, myoclonus, muscular atrophy, or hypertrophy was observed. The remaining physical examination was unremarkable. Laboratory studies were notable for hypercholesterolemia (total cholesterol 312 mg/dL), impaired renal function (creatinine 1.5 mg/dL), and marked elevation of muscle enzymes, including CK (5178 U/L), myoglobin (158 ng/mL), lactate dehydrogenase (864 U/L), aspartate transaminase (82 U/L), and alanine transaminase (43 U/L) (Table 1). Urinalysis showed moderate occult blood, without dysmorphic red blood cells on microscopic examination, and urine protein to creatinine ratio was 0.05 g/g. Abdominal sonography revealed no evidence of hydronephrosis.

**TABLE 1:** Laboratory data.

Variable	Unit	Reference	Local clinic	On admission to this hospital	Three months after discharge	Nine months after discharge
Sodium	mmol/L	136–145	138	137	137	
Potassium	mmol/L	3.5–5.1	4.5	3.9	3.7	
Chloride	mmol/L	98–107		98		
Carbon dioxide	mmol/L	22–29		25		
Total calcium	mg/dL	8.6–10.2		9.8		
Phosphorus	mg/dL	2.7–4.5		4.4		
Magnesium	mg/dL	1.7–2.6		2.1		
BUN	mg/dL	6–20	22	26	19	13
Creatinine	mg/dL	0.6–0.9	0.9	1.5	0.9	0.8
AST	U/L	9–32	26	82	18	13
ALT	U/L	7–31	18	43	21	8
CK	U/L	26–308		5,178	118	
LDH	U/L	135–225		864		
Myoglobin	ng/mL	< 110		158		
Fasting glucose	mg/dL	74–109	78	98		89
Uric acid	mg/dL	2.4–5.7	5.9	6.2	5.8	7.6
Total cholesterol	mg/dL	< 200	345	312	169	189
Triglyceride	mg/dL	< 200	244	195	187	93
LDL-C	mg/dL	< 100	266	235	110	119
HDL-C	mg/dL	> 40	69	65	48	57
T3	ng/dL	86–187		33.99		
TSH	μIU/mL	0.25–5.0		> 45	4.3	3.9
Free T4	ng/dL	0.8–2.0		< 0.2	0.9	1.1

ALT, Alanine aminotransferase; AST, Aspartate aminotransferase; BUN, blood urea nitrogen; CK, creatine kinase; HDL-C, high-density lipoprotein cholesterol; LDH, lactate dehydrogenase; LDL-C, low-density lipoprotein cholesterol; TSH, thyroid-stimulating hormone.

**FIGURE 1:**
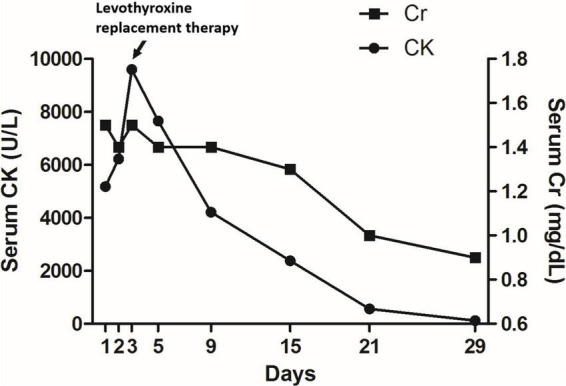
Changes in serum CK levels and renal function after presentation. CK, creatine kinase; Cr, creatinine.

During the hospitalization, the patient did not have hypotension, signs of infection, or heart failure. Due to the presumptive diagnosis of statin-induced rhabdomyolysis, atorvastatin was discontinued. Despite intravenous volume repletion (isotonic saline 2 L/day) and alkalinization (sodium bicarbonate 64 mEq/day), her myalgia persisted, serum CK level increased to 9,594 U/L, and creatinine level remained at 1.5 mg/dL 2 days later. An investigation for other causes of rhabdomyolysis revealed low T3 (33.99 ng/dL, reference change 86–187), low free T4 (<0.2 ng/dL, reference change 0.8–2.0), and high thyroid-stimulating hormone (TSH) (> 45 μIU/mL, reference change 0.25–5.0), consistent with hypothyroidism. Other serological tests for autoimmune diseases, including antinuclear antibody, rheumatoid factor, anti-double-stranded DNA, and anti-Ro/La showed normal results. Tests to determine the etiology of hypothyroidism showed the presence of anti-thyroperoxidase and anti-TSH receptor antibodies. A neck ultrasound showed reduced echogenicity of the thyroid. Based on clinical, laboratory, and imaging findings, a diagnosis of Hashimoto’s thyroiditis was made. After thyroid hormone replacement with thyroxine 200 μg daily for 1 month, the patient’s clinical symptoms resolved, and serum CK and creatinine levels returned to the normal range (Figure 1). Three months later, restoration of euthyroidism was achieved, and no recurrence of elevated serum CK or muscular sequelae was observed. Her lipid profile was within normal range, and atorvastatin was discontinued. Six months following the discontinuation of the atorvastatin, her total cholesterol level was 189 mg/dL, and her triglyceride level was 93 mg/dL.

## Discussion

This woman on atorvastatin for her hypercholesterolemia for 1 month exhibited flank rhabdomyolysis and acute kidney injury with the presumptive diagnosis of statin-induced myopathy. According to her hormone levels, she had unnoticed hypothyroidism. With thyroxine replacement, her clinical symptoms rapidly resolved along with the normalization of serum CK and creatinine levels, supporting the diagnosis of hypothyroidism-induced rhabdomyolysis and acute kidney injury.

The classic symptoms and signs related to hypothyroidism, such as fatigue, cold intolerance, weight gain, constipation, hoarse voice, puffy face, peripheral edema, and dry, coarse skin, may be absent or subtle and also frequently overlooked. Early diagnosis of hypothyroidism is often difficult. However, hypercholesterolemia is a very common and even sole biochemical finding in hypothyroidism due to its effect on a reduction in LDL receptors, increase in intestinal cholesterol absorption, decrease in cholesteryl ester transfer protein, and decrease in hepatic lipase. Unlike low HDL-C in primary hypercholesterolemia, a normal to higher HDL-C level is also a landmark of hypothyroidism (2). Of note, hypercholesterolemia in hypothyroidism is often resistant to statin therapy, as shown in this case. It is thus suggested that thyroid function should be examined in all patients with severe hypercholesterolemia before lipid-lowering therapy (3).

Similar to statins, hypothyroidism per se can also cause various muscle disorders, including rhabdomyolysis. Because muscular symptoms are common in hypothyroidism, hypothyroid myopathy is referred to patients with a clinical picture of predominant muscular involvement in the absence of the classic presentation of hypothyroidism. A total of 20–80% of hypothyroid patients have symptoms and signs of hypothyroid myopathy. Rhabdomyolysis due to hypothyroidism is not rare, with an unknown prevalence (4). The causative mechanism behind myopathy in hypothyroidism remains elusive. Inhibition of mitochondrial activity and altered metabolic pathways in muscle cells may be involved in this process (4). The comparison between statin-induced myopathy and hypothyroidism myopathy is shown in Table 2. Although statin and hypothyroid myopathy have similar clinical presentations, myoedema and slowed movements and reflexes are present only in hypothyroidism. Despite no myoedema, the slow relaxation of deep tendon reflexes in this patient suggested that myopathy was caused by hypothyroidism rather than statin. We speculated that the initiation of atorvastatin might precipitate preexisting hypothyroid myopathy, leading to severe rhabdomyolysis.

**TABLE 2:** Difference between statin and hypothyroid myopathy.

	Statin-related myopathy	Hypothyroid myopathy
Prevalence	10–25% on statin therapy	20–80% in overt hypothyroidism
Risk factors	High dose of statin, drugs affecting statin metabolism, old age, hypothyroidism, low body mass index, female sex	Diabetes mellitus, liver and renal disease, alcoholism, lipid-lowering drugs, strenuous exercise, old age
Possible mechanisms	Reduced sarcolemmal or tubule cholesterol coenzyme Q10 depletion	Impaired glycogenolysis and mitochondrial oxidative metabolism
Clinical presentations	Pain, weakness and sometimes muscle swelling	Pain, proximal weakness, myoedema slowed movements and reflexes
Rhabdomyolysis	1 case/10,000 person-years	Not uncommon
Renal function	Usually normal	Often reduced (reduced renal blood flow)
Management	Statin withdrawal Urine alkalinization for rhabdomyolysis	Thyroxine replacement Urine alkalinization for rhabdomyolysis

To date, flank rhabdomyolysis associated with undiagnosed hypothyroidism has been reported in more than 24 patients on statin therapy in the literature (5–18). These patients were characterized by male predominance (13 patients), older age (> 50 years), prominent hypercholesterolemia, development of muscle symptoms days to weeks after starting statin therapy, and resolution of rhabdomyolysis with the correction of hypothyroidism. Among them, 10 patients were complicated with acute kidney injury, and two patients required renal replacement therapy. However, all of them had their renal function recovered within several days to 2 months.

It is also well-documented but still less appreciated that hypothyroidism *per se* can lead to abnormal renal function (acute kidney injury) via reduced cardiac output, increased systemic and renal vascular resistance, and a resultant reduction in glomerular filtration rate. Acute kidney injury may also be caused by concomitant rhabdomyolysis through intra-renal vasoconstriction, direct ischemic tubular injury, and tubular obstruction. Some crucial points on rhabdomyolysis-induced acute kidney injury should be emphasized. First, rhabdomyolysis-induced acute kidney injury frequently causes oliguria and often leads to a more rapid increase in serum creatinine (19). Second, the risk of acute kidney injury in non-traumatic rhabdomyolysis remains low if serum CK at presentation is less than 15,000–20,000 U/L (20). Third, physicians must search for the coexisting conditions that can cause acute kidney injury in patients without severe rhabdomyolysis. The chronically increased serum CK levels without a remarkable rise in serum creatinine and oliguria made rhabdomyolysis-induced acute kidney injury less likely. Normalized serum creatinine and CPK level following the treatment of hypothyroidism confirmed that hypothyroidism was the primary cause of reduced renal function and rhabdomyolysis.

In conclusion, unnoticed hypothyroidism can cause concomitant rhabdomyolysis and kidney injury, especially in patients on statin therapy for hypercholesterolemia. Our case also highlights the importance of keeping hypothyroidism in mind as a diagnosis in patients with statin-resistant hypercholesterolemia, unexplained abnormal renal function, and/or rhabdomyolysis. Prompt recognition of hypothyroidism with thyroxine replacement avoids unnecessary administration of statins and other potential complications.

## Data availability statement

The raw data supporting the conclusions of this article will be made available by the authors, without undue reservation.

## Author contributions

W-FC wrote the article. J-SC played an important role in the analysis and interpretation of the data. P-JH made the draft of the manuscript. S-HL took responsibility for the work as a whole and approved the final submitted version of the manuscript. All authors contributed to the article and approved the submitted version.

## References

[1] Thompson PD Panza G Zaleski A Taylor B. Statin-associated side effects. *J Am Coll Cardiol.* (2016) 67:2395–410. 10.1016/j.jacc.2016.02.071 27199064

[2] Pearce EN. Update in lipid alterations in subclinical hypothyroidism. *J Clin Endocrinol Metab.* (2012) 97:326–33. 10.1210/jc.2011-2532 22205712

[3] Jellinger PS Handelsman Y Rosenblit PD Bloomgarden ZT Fonseca VA Garber AJ American association of clinical endocrinologists and American college of endocrinology guidelines for management of dyslipidemia and prevention of cardiovascular disease. *Endocr Pract.* (2017) 23:1–87. 10.4158/EP171764.APPGL 28437620

[4] Sindoni A Rodolico C Pappalardo MA Portaro S Benvenga S. Hypothyroid myopathy: a peculiar clinical presentation of thyroid failure. Review of the literature. *Rev Endocr Metab Disord.* (2016) 17:499–519. 10.1007/s11154-016-9357-0 27154040

[5] Al-Jubouri MA Briston PG Sinclair D Chinn RH Young RM. Myxoedema revealed by simvastatin induced myopathy. *BMJ.* (1994) 308:588. 10.1136/bmj.308.6928.588 PMC2539565 8148686

[6] Ahmad S. Lovastatin-induced myopathy in a hypothyroid patient. *J Fam Pract.* (1995) 41:227–8. 7650497

[7] Hung YT Yeung VT. Hypothyroidism presenting as hypercholesterolaemia and simvastatin-induced myositis. *Hong Kong Med J.* (2000) 6:423–4. 11177166

[8] Gemici G Toprak A Oktay A. Rhabdomyolysis due to cerivastatin monotherapy. *Am J Med.* (2001) 110:742. 10.1016/s0002-9343(01)00730-6 11417566

[9] Rando LP Cording SA Newnham HH. Successful reintroduction of statin therapy after myositis: was there another cause?. *Med J Aust.* (2004) 180:472–3. 15115429 10.5694/j.1326-5377.2004.tb06030.x

[10] Tokinaga K Oeda T Suzuki Y Matsushima Y. HMG-CoA reductase inhibitors (statins) might cause high elevations of creatine phosphokinase (CK) in patients with unnoticed hypothyroidism. *Endocr J.* (2006) 53:401–5. 16723812 10.1507/endocrj.k04-144

[11] Bar SL Holmes DT Frohlich J. Asymptomatic hypothyroidism and statin-induced myopathy. *Can Fam Physician.* (2007) 53:428–31. 17872677 PMC1949076

[12] Hilton-Jones D. Myopathy associated with statin therapy. *Neuromuscul Disord.* (2008) 18:97–8. 10.1016/j.nmd.2007.08.008 17892936

[13] Krieger EV Knopp RH. Hypothyroidism misdiagnosed as statin intolerance. *Ann Intern Med.* (2009) 151:72. 10.7326/0003-4819-151-1-200907070-00018 19581652

[14] Qari FA. Severe rhabdomyolysis and acute renal failure secondary to use of simvastatin in undiagnosed hypothyroidism. *Saudi J Kidney Dis Transpl.* (2009) 20:127–9. 19112232

[15] Ambapkar SN Shetty N Dwivedy A Malve HO. Statin-induced rhabdomyolysis in patient with renal failure and underlying undiagnosed hypothyroidism. *Indian J Crit Care Med.* (2016) 20:305–7. 10.4103/0972-5229.182210 27275082 PMC4876655

[16] Ahn P Min HJ Park SH Lee BM Choi MJ Yoon JW Rhabdomyolysis and acute kidney injury associated with hypothyroidism and statin therapy. *Endocrinol Metab.* (2013) 28:331–4. 10.3803/EnM.2013.28.4.331 24396699 PMC3871041

[17] Ram R Swarnalatha G Ramesh V Rao KN Dakshinamurty KV. Rhabdomyolysis induced acute renal failure secondary to statins. *Indian J Nephrol.* (2013) 23:211–3. 10.4103/0971-4065.111853 23814421 PMC3692148

[18] Peringat J Manappallil RG Karadan U. Rhabdomyolysis: a rare complication of hashimoto’s thyroiditis precipitated by statin therapy. *BMJ Case Rep.* (2018) 2018:bcr2017223229. 10.1136/bcr-2017-223229 29440138 PMC5836711

[19] Bosch X Poch E Grau JM. Rhabdomyolysis and acute kidney injury. *N Engl J Med.* (2009) 361:62–72. 10.1056/NEJMra0801327 19571284

[20] Veenstra J Smit WM Krediet RT Arisz L. Relationship between elevated creatine phosphokinase and the clinical spectrum of rhabdomyolysis. *Nephrol Dial Transplant.* (1994) 9:637–41. 7970089 10.1093/ndt/9.6.637

